# The influence of different sample preparation on mechanical properties of human iliotibial tract

**DOI:** 10.1038/s41598-020-71790-5

**Published:** 2020-09-09

**Authors:** Benjamin Fischer, Sascha Kurz, Andreas Höch, Stefan Schleifenbaum

**Affiliations:** 1 ZESBO - Center for Research On the Musculoskeletal System, Leipzig University, Semmelweisstraße 14, 04103 Leipzig, Germany; 2 Department of Orthopedic, Trauma and Plastic Surgery, Spine Center, Leipzig University, Leipzig, Germany; 3 Fraunhofer Institute for Machine Tools and Forming Technology, Chemnitz, Germany

**Keywords:** Materials science, Anatomy, Musculoskeletal system, Biological physics

## Abstract

In the run-up to biomechanical testing, fresh human tissue samples are often frozen in order to inhibit initial decomposition processes and to achieve a temporal independence of tissue acquisition from biomechanical testing. The aim of this study was to compare the mechanical properties of fresh tissue samples of the human iliotibial tract (IT) to fresh-frozen samples taken from the same IT and those modified with different concentrations of Dimethylsulfoxide (DMSO) prior to freezing. All samples were partial plastinated and destructive tensile tests were conducted with a uniaxial tensile test setup. A plastination technique already established in the laboratory was modified to improve the clamping behaviour of the samples. Material failure was caused by a gradual rupture of the load-bearing collagen fibre bundles. Contrary to our expectations, no significant difference was found between the tensile strength of fresh and fresh frozen specimens. The addition of 1 wt% DMSO did not increase the tensile strength compared to fresh-frozen samples; an addition of 10 wt% DMSO even resulted in a decrease. Based on our findings, the use of simple fresh-frozen specimens to determine the tensile strength is viable; however fresh specimens should be used to generate a complete property profile.

## Introduction

Tissue stored in fresh frozen condition is preferred for biomechanical testing of human soft tissue. This allows decoupling tissue acquisition, which usually has to be done shortly after donor death, and material testing. Especially in case of ligaments and aponeuroses, the influence of freezing and thawing processes on the mechanical properties of human tissue has not yet been fully investigated, despite current research in this field^1–5^. There is a large number of different research results in the field of biomechanical characterization of ligaments and tendons, particularly with regard to the influence of donor age and the influence of freezing effects on the mechanical properties^6–11^. The formation of ice crystals of varying size in intra- and extracellular water during the freezing process is known to cause tissue damage and is associated with penetration of cell membranes and extracellular structures.

In cell culture laboratories, various antifreeze agents such as dimethyl sulfoxide (DMSO) and glycerin are used to prevent tissue damage at the cellular level during deep-freeze storage. The aim of this study was to investigate the influence of freezing and thawing processes on the deformation behaviour and mechanical properties of the human iliotibial tract (IT) compared to the fresh state. The mechanical properties of fresh-frozen tissue pre-treated with DMSO were also investigated. The use of tissue in as fresh a condition as possible is a distinguishing feature of this study, which served as the basic tissue condition for the differently prepared sample variants. The IT was chosen for this study, as it has been extensively studied in the past^12–19^. Additionally, due to the size of this structure, sample acquisition is relatively simple and allows the extraction of several samples from individual IT, thus improving comparability.

## Methods

### Sample preparation

Twenty fresh tissue samples of human IT from ten body donors (six male and four female) were prepared.

All donors originated from the Institute of Anatomy of the Leipzig University and had given written consent to dedicate their bodies to medical education and research purposes. Being part of the body donor program regulated by the Saxonian Death and Funeral Act of 1994 (3rd section, paragraph 18, item 8), institutional approval for the use of the post-mortem tissues of human body donors was obtained. The authors declare that all experiments were performed according to the ethical principles of the Declaration of Helsinki.

**Figure 1:**
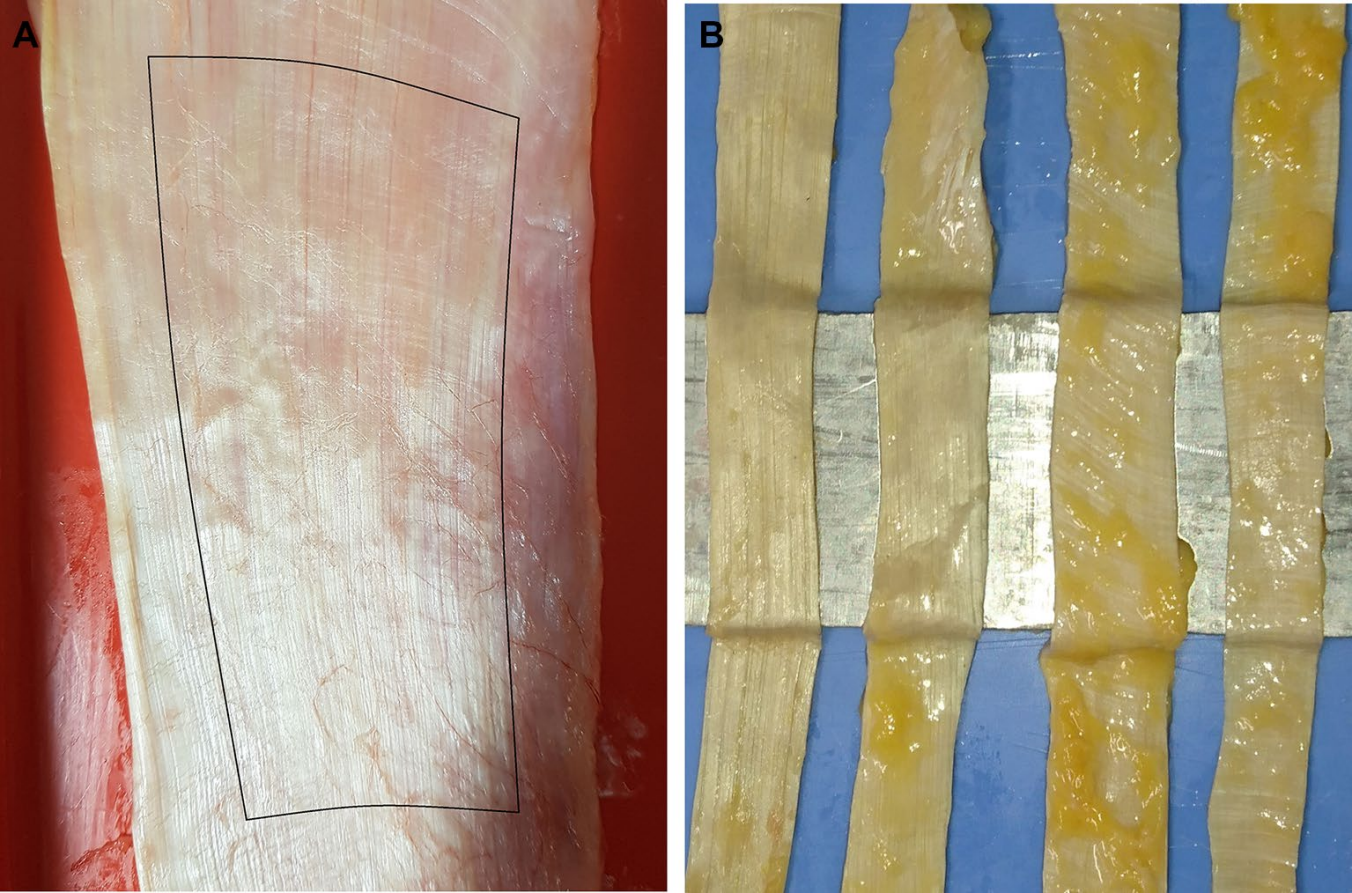
**Left:** Harvested IT with extraction area of the four samples (black outline). The curvature is a result of the placement of the iliotibial tract; **Right**: Extracted samples prior to plastination.

The mean donor age was 82.7 (SD 8.79) years, mean donor body mass was 73.2 (SD 17.78) kg. With a mean donor size of 1.68 (SD 0.09) m, the mean body mass index (BMI) was 25.73 (SD 5.18) kg/m^2^. The parallel main fibre orientation of the collagenous structure was determined visually. Each sample was cut lengthwise into four rectangular parts, according to the orientation of the load bearing collagen fibres. The upper and the lower part of the IT were removed first with a scalpel. The rectangular strips were manually cut by following the dominant fibre bundles with a scalpel until the upper and lower end of the extracted IT sample was reached (Fig. 1).

The widths of all specimen was documented with a mean width of 1.13 mm. A mean thickness of 0.9 mm was determined for all specimen. Each of the four parts was assigned to a different preparatory procedure prior to testing (Table 1). Samples prepared for unmodified deep freeze storage (Type II) were placed in physiological saline solution (PSS). For the treatment of sample types III and IV, DMSO in American Chemical Society purity grade (ACS) was used (Merck KGaA, Darmstadt, Deutschland) in combination with PSS.

**Table 1:** Sample preparation of the four different sample states: Fresh (F), Fresh-deep-frozen (FDF0), Fresh-frozen with 1 wt% DMSO (FDF1) and Fresh-frozen with 10 wt% DMSO (FDF10).

Type	Abbreviation	Sample preparation
I	F	Fresh sample
II	FDF0	Storage in PSS (20 min) before freezing
III	FDF1	Storage in PSS with 1 wt% DMSO (20 min) before freezing
IV	FDF10	Storage in PSS with 10 wt% DMSO (20 min) before freezing

**Figure 2:**
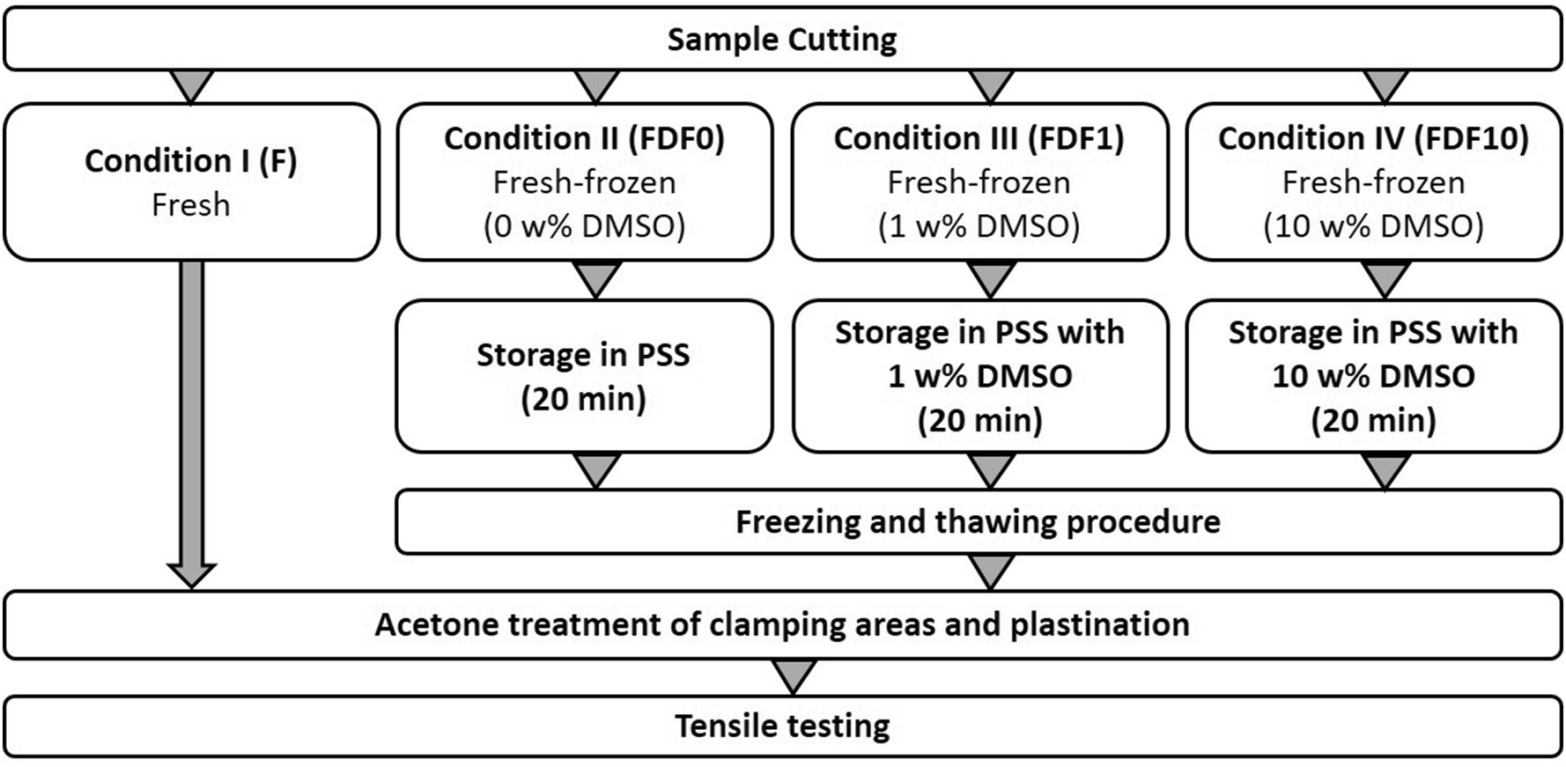
Scheme of sample preparation.

In this study we used an already established freezing method and exposed tissue samples to different concentrations of DMSO. The freezing regime for sample categories II–IV after storage in the respective solution comprised four temperature levels. Starting from room temperature (20 °C), the samples were stored at 5 °C (30 min) in a first step, then at -20 °C (30 min) and finally at -80 °C. The further workflow of the preparation is shown in Fig. 2.

Fresh specimens were placed between two congruent aluminium plates, clamped and prepared for partial plastination. These plates had the purpose to shield the test areas of the specimens from the acetone during the subsequent dehydration step of the clamping zones. The length of the test zones was standardized to 50 mm.

**Figure 3:**
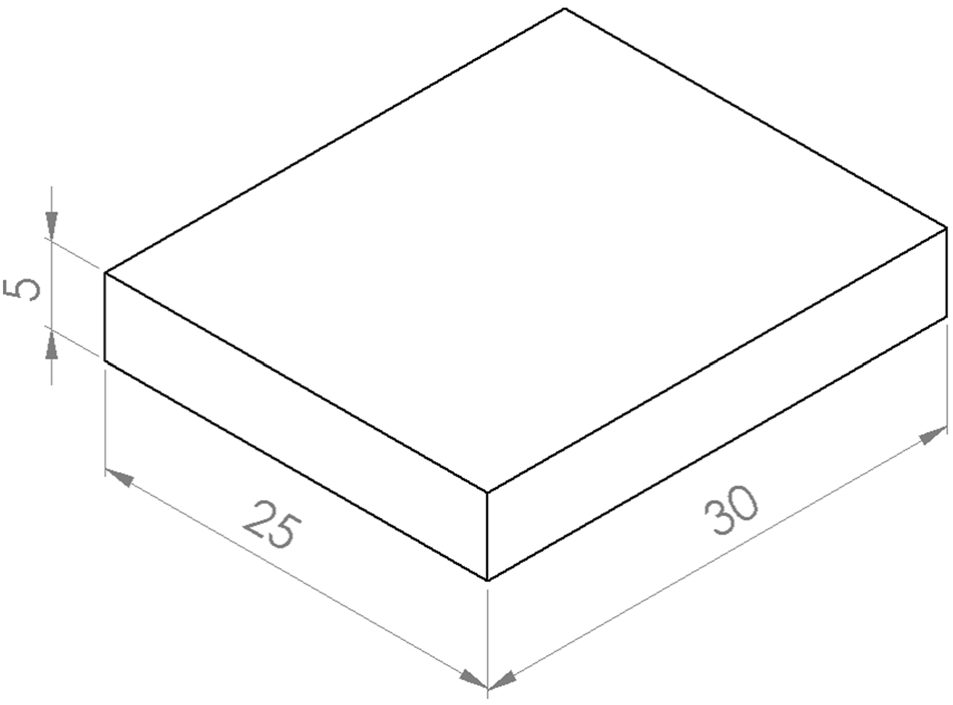
Dimensions of the beech wood panels used (5 × 25 × 30 (H × W × D) mm).

To create a better contact zone between the samples and the applied resin, the plastination areas were immersed in pure acetone (J. T. Baker, Avantor, VWR International Global Exports, Radnor, PA, USA) for 20 min per side to degrease and dry the clamp areas. For plastination, two beech wood panels of congruent shape were sawn for each clamping area (Fig. 3).

The parts have been designed to provide maximum contact with the jaws of the Instron 5566A (Instron Corporation, Norwood, MA, USA) table-top testing machine. The resin used in this partial plastination technique was a two-component polyurethane rapid casting resin (RenCast FC 52/53 isocyanate/FC 52 polyol; Huntsman Advanced Materials, East Lansing, MI, USA) filled with aluminium hydroxide powder (Gössl + Pfaff GmbH, Karlskron, Deutschland). The mixing ratio was 1:1:3 (Isocyanate:Polyol:Al(OH)_3_). After mixing and thorough stirring, the resin-powder mixture was split into kneadable portions which were placed tightly around the dried clamping areas of the samples to ensure central placement. To increase the pull-out strength, the samples were additionally folded in the resin. The pre-cut pieces of beech wood were placed above and below before the complete stack was clamped for 30 min. In the last step, the aluminium plates which protected the fresh test areas of the sample were removed from acetone and the resin mixture. Immediately afterwards the testing was carried out.

Due to the fact that only complete sample series of states I–IV were considered for the final testing and evaluation, singular samples had to be discarded.

### Testing

The tensile tests were performed on an Instron 5566A table-top testing machine using a uniaxial tensile test method and a 1 kN load cell. A low preload of 10 N was chosen prior to the tensile testing process in order to ensure alignment of the collagen fibres and thus to achieve easy preconditioning. As there is no ideal testing norm for the testing of human aponeurotic tissue, the test speed was based on the ISO 13934-1, which recommends a strain rate of 0.1 mm per mm sample length per minute for an expected failure strain of < 10%, which we expected here. Thus deformation was performed at a speed of 5 mm/min for our specimen length of 50 mm. The abort criterion of the procedure was a reduction in tensile stress by 30%, relative to the maximum value. The recording of the measured values was path-controlled. Force and displacement data were processed with the Bluehill 2 Software (Instron Corporation, Norwood, MA, USA). Engineering stress and engineering strain values were calculated based on this data using Microsoft Excel (Microsoft Corporation, Redmond, WA, USA). The Young’s Modulus was calculated in the linear part of the stress–strain curves with E = (σ_40%_ − s_20%_)/(ε_40%_ − ε_20%_), with σ_20%_ and σ_40%_ being the stress values at 20% and 40% of the yield strength. Statistical analysis was performed with SPSS (IBM, Armonk, NY, USA) utilizing the Paired t Test.

## Results

### Sample preparation

**Figure 4:**
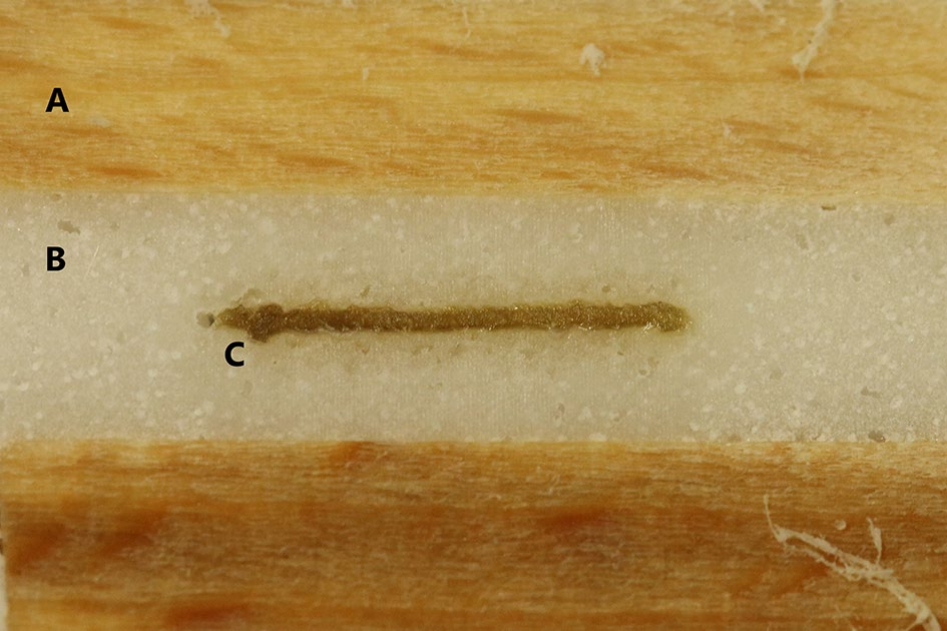
Cross section through the plastinated clamping zone. A conclusive material bond between wood (**A**), resin-hydroxide mixture (**B**) and plane-parallel embedded iliotibial tract specimen can be recognized (**C**).

The modified partial plastination process of the specimen clamping areas was successful for most samples. Two samples had to be discarded due to an inhomogenous resin mixture, which lead to insufficient processing properties of the resin-hydroxide-mix. Besides these two cases, the resin-hydroxide composite infiltrated the tissue and the beech wood equally and created a stable and firm connection between the specimen and the clamping material, as examined and determined in cross sections through the clamping zones (Fig. 4).

Some specimens had to be discarded due to dehydration and faulty sample cutting, which is why only complete specimen groups (states I, II, III & IV) were included in the evaluation.

### Testing

**Figure 5:**
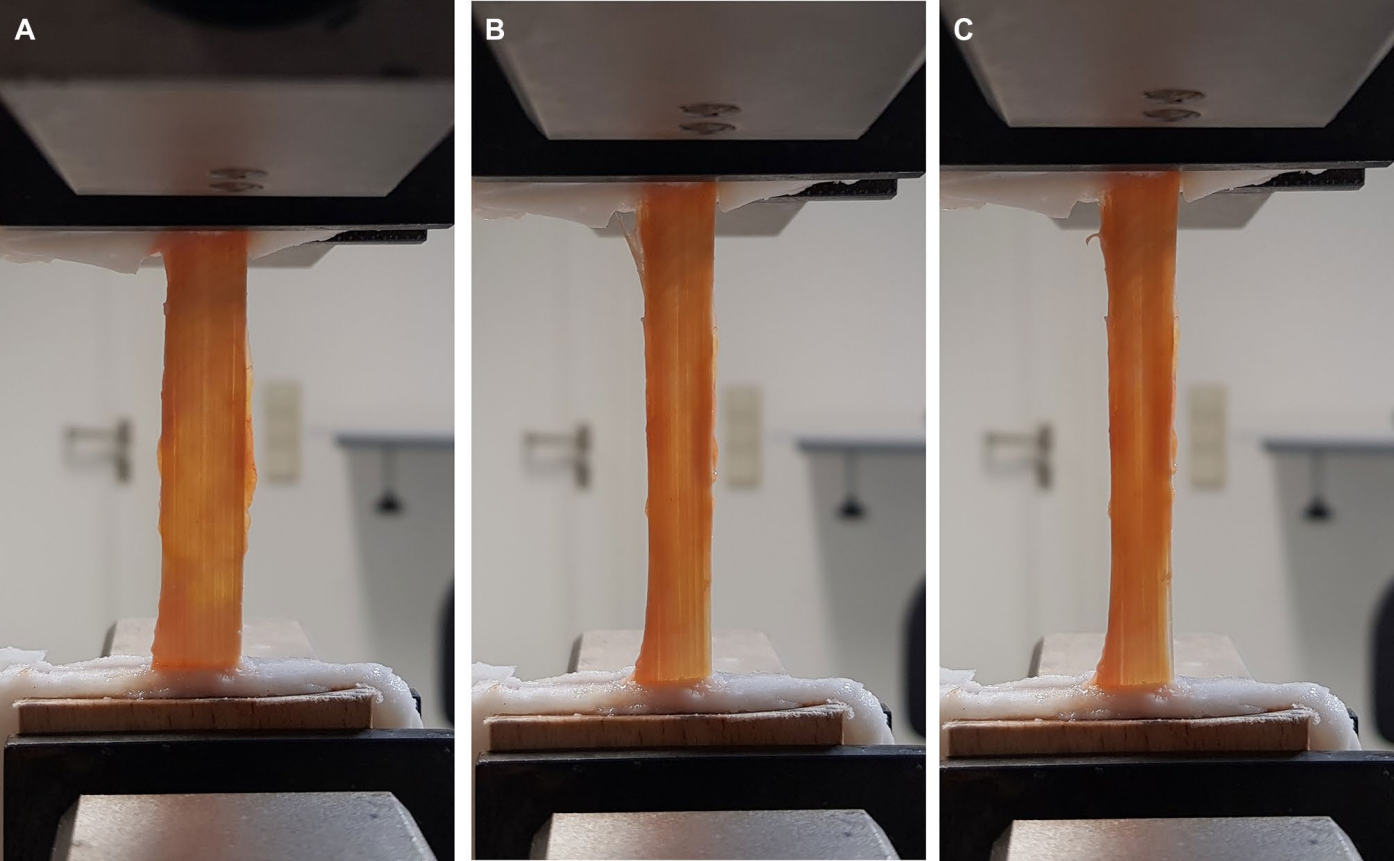
Procedure for uniaxial tensile testing. **Left**: Under preload. Clearly visible at the edges left and right of the test area is the superficial tissue layer, which is not load bearing and masks the collagen fibre bundles, **Mid**: Immediately before failure, **Right**: After exceeding tensile strength and incipient failure of collagen fibre bundles at the bottom right area.

In contrast to the classical tensile test, the material failure did not occur by an abrupt and complete tearing of the specimen, but by a gradual failure of the load-bearing collagen fibre bundles (Fig. 5). Two samples were affected by the problem of slippage, which is known to occur in this type of tissue ^18^.

Specimen failure occurred with considerable donor-specific differences between 5.6 and 61.6 MPa; the mean values and moduli of elasticity determined are listed in Table 2.

**Table 2:** Determined average tensile strength (F_tu_) and Young’s Modulus (E) for the sample states F, FDF0, FDF1 and FDF10.

	F	FDF0	FDF1	FDF10
F_tu_ (MPa)	37.66 (SD 18.87)	34.64 (SD 12.08)	30.96 (SD 13.12)	22.00 (SD 10.91)
E (MPa)	0.73 (SD 0.44)	0.91 (SD 0.29)	0.83 (SD 0.28)	0.65 (SD 0.28)

**Figure 6:**
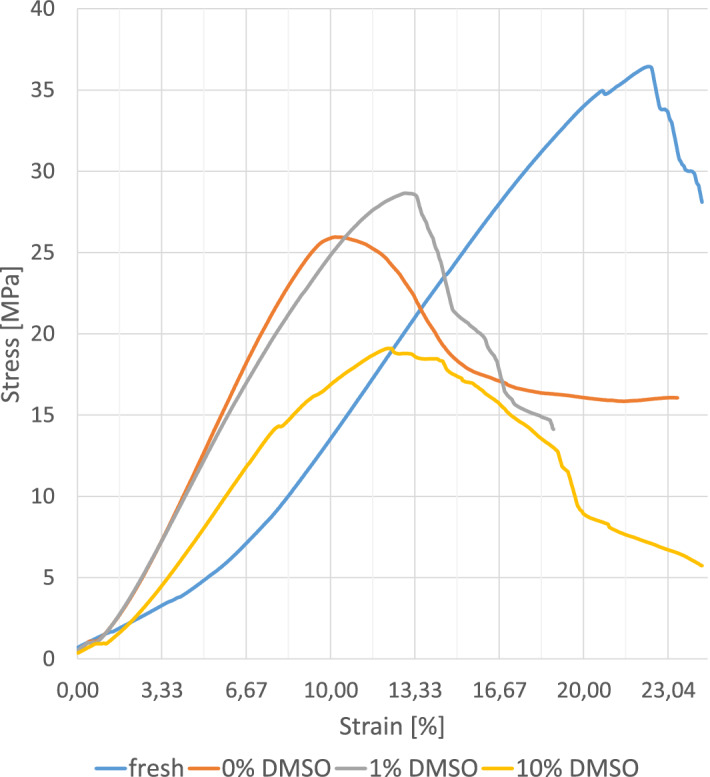
Stress–Strain-curves of different sample types: fresh (I), 0% DMSO (II), 1% DMSO (III) and 10% DMSO (IV).

**Figure 7:**
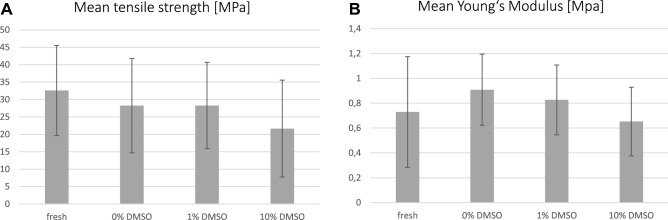
Mean tensile strength (MPa) and Mean Young’s Modulus (MPa).

Non-significant differences with slightly decreasing tensile strength values between the state F samples (37.66 (SD 18.87) MPa), FDF0 samples (34.64 (SD 12.08)) MPA) and FDF1 samples (30.96 (SD 13.12)) MPa) were determined, while the strengths of FDF10 samples were significantly lower (22.00 (SD 10.91) MPa). An exemplary stress–strain curve can be seen in Fig. 6. A slight increase in the Young’s Modulus of FDF0 samples (0.91 (SD 0.29) MPa) and FDF1 samples (0.83 (SD 0.28) MPa), compared to F samples (0.73 (SD 0.44) MPa) has been observed (Fig. 7). With a total of 10 samples (5 body donors), the entire process chain from cutting to size to plastination and final testing was successful, however three samples had to be excluded from the calculation of the mean ultimate tensile strength, as sample failure occurred close to the clamping area.

A significant decrease in tensile strength has been observed between the fresh state F (I) and the FDF10 state (IV) (p = 0.017), and between the FDF0 state (II) and the FDF10 samples (IV) (p = 0.006). No significant correlations were found for the comparison of the states F (I) with FDF0 (II) (p = 0.570), fresh (I) with FDF1 (III) (p = 0.432) and FDF0 (II) with FDF1 (III) (p = 0.065).

## Discussion

The partial plastination technique used by Steinke et al.^15^ was modified to further minimize the slippage of the tissue samples. The use of wooden plates in the plastination process instead of polymer plates was advantageous for several reasons. The clamping behaviour improved because the metal clamps of the testing machine were able to penetrate the wood material and prevent the clamping areas from slipping. In addition, the resin was partially absorbed and integrated into the surficial fibre structure of the wood, resulting in a much stronger bond between the resin layer and the wood panels. The possibility of folding the tissue samples due to their long geometry probably also increased the pull-out strength and caused the reduced occurrence of slippage effects. However, this has to be verified in further experiments with the use of digital image correlation systems.

An essential difference in the IT investigated here compared to classical materials characterized by uniaxial tensile tests is the completely different failure pattern. An abrupt rupture (commonly observed in ceramic, brittle polymeric and metallic test specimens) or a characteristic failure pattern, which is typical for elastic polymers, was not observed. The material failure occurred by a cascading rupture of the load-bearing collagen fibre bundles, which manifested itself in gradual tension drops after exceeding the tensile strength. A similar failure behaviour can be found, for example, in the staggered rupture of a steel cable or braided rope, where the individual elements of the overall structure also fail in succession. In previously published studies on uniaxial tensile testing of ligaments and tendons, an hourglass shape is often chosen for the test areas, analogous to tensile test specimens made of metallic materials^11,20^. However, we deliberately chose a rectangular shape in order to reduce the number of interrupted collagen fibrils as much as possible, as these could in principle serve as initial defects for the material failure. The main objective was to obtain long strips of tissue with edges parallel to the internal, load bearing collagen structure, which has been used in similar studies^20–22^. This also assured that the direction of the tensile tests was colinear to this load bearing collagen structure. A final, comparative evaluation of the ideal sample shape should be made in further investigations, especially with regard to the staggered sample failure pattern.

We found in preliminary tests that the deformation behaviour of the load bearing structure was not completely reflected by the superficial, visible connective tissue layer, but was masked due to slipping of the tissue layers during testing. Thus, we did not use digital image correlation. Solving this problem could be content of a future study.

Contrary to our expectations, no significant difference was found between the tensile strength of fresh (I) and fresh frozen (II) specimens. Possibly the careful freezing was a partial cause for this, as the method used was optimized by a special cooling regime to minimize ice crystal growth in the tissue samples.

In this context, earlier studies by Lansdown et al. for ligament structures showed a negligible influence of freezing processes on the mechanical properties of human cruciate ligaments. Freezing–thawing cycles of up to eight times did not lead to any significant changes in strength^23^. This is consistent with our observations that, contrary to our initial hypothesis, the mean strength parameters of fresh, fresh-frozen and acetone-treated frozen iliotibial tract samples were in similar ranges.

Hochstraht and Müller et al. performed investigations on mouse tendons and compared on a microscopic level the condition of fresh, phosphate-buffered saline solution (PBS) treated and frozen as well as DMSO treated and frozen Achilles tendons. A change in the collagen fibre orientation due to freezing damage was observed at the molecular level, which is consistent with our hypothesis. In the biomechanical tests performed by Hochstraht and Müller et al., a significant correlation between the number of freezing cycles and the increase in elastic modulus was found. The described reduction of the static Young’s Modulus in samples frozen once is comparable to our results^24^.

A decrease in the tensile strength between group I and IV in our experiment could be attributed to a reduction in the collagen strength of the samples exposed to higher DMSO values. A splitting of the intermolecular cross-linking of collagen fibres is the most plausible cause, as Gries et al. showed^25^. An increase of the DMSO level could therefore reinforce this effect and lead to an earlier material failure of the load-bearing structure. However, despite their very similar molecular composition, the tissue samples in the mentioned publications were tissue from different species. The biomechanical test conditions also varied.

We deliberately refrained from preconditioning in order to prevent tissue damage in advance of the destructive material test. The reason for that was to be able to investigate the effects of the different sample preparation methods on the mechanical tissue properties on the tensile strength and the Young’s Modulus in as isolated a manner as possible.

### Limitation

The limited number of samples due to the requirement to examine fresh donor tissue must be taken into account with regard to the results obtained. Donor-specific differences are therefore more impactful, compared to a larger sample size. In further investigations, the use of a digital image correlation technique is also useful to verify the quality of the slip reduction achieved by the modified plastination technique and to determine the detailed sample failure pattern. However, this could also lead to a deterioration in the visibility of the staggered sample failure, which would have to be assessed.

## Conclusion

Non-significant differences with slightly decreasing tensile strength values of fresh (I), fresh-frozen samples without DMSO addition (II) and fresh-frozen samples with 1% DMSO addition (III) were determined. In contrast the tensile strengths of fresh-frozen samples with 10% DMSO addition were significantly lower. DMSO could to be responsible for the splitting of the crosslinks between the collagenous fibres, but this has to be proven in further studies. No positive effect on the mechanical properties of DMSO in cryopreservation was found. Likewise, no significant negative effect of freezing on tensile strength could be observed with single freeze–thaw cycles. On the other hand, a slight increase in the Young’s Modulus has been observed. The use of simple fresh-frozen specimens to determine the tensile strength can therefore be regarded as legitimate; fresh specimens should be used to establish a complete property profile including the Young's modulus, if possible.
