# Author Correction: The influence of different sample preparation on mechanical properties of human iliotibial tract

**DOI:** 10.1038/s41598-021-81980-4

**Published:** 2021-01-25

**Authors:** Benjamin Fischer, Sascha Kurz, Andreas Höch, Stefan Schleifenbaum

**Affiliations:** 1 ZESBO - Center for Research on the Musculoskeletal System, Leipzig University, Semmelweisstraße 14, 04103 Leipzig, Germany; 2 Department of Orthopedic, Trauma and Plastic Surgery, Spine Center, Leipzig University, Leipzig, Germany; 3 Fraunhofer Institute for Machine Tools and Forming Technology, Chemnitz, Germany

Correction to: *Scientific Reports* 10.1038/s41598-020-71790-5, published online 09 September 2020

This Article contains errors in Figure 7. The correct Figure 7 appears below as Figure 1.

**Figure 1:**
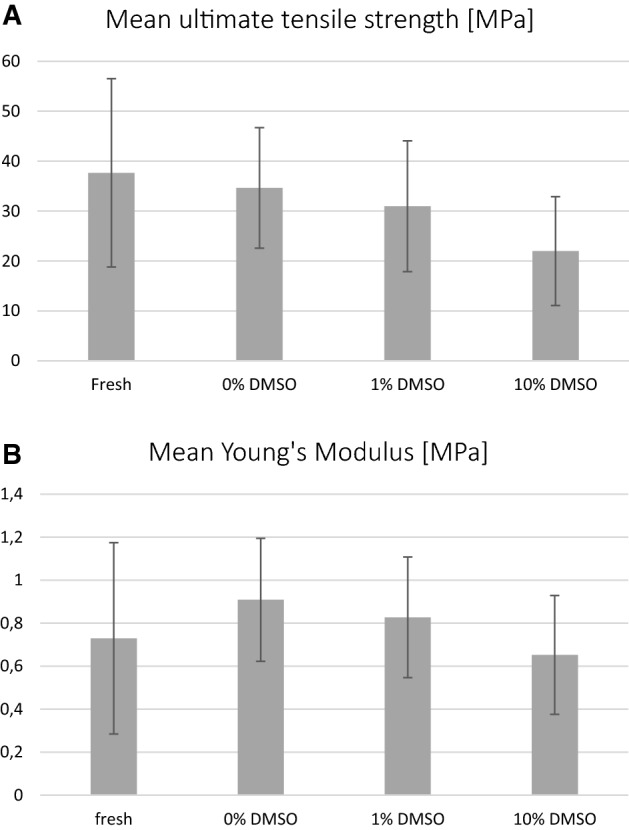
A correct version of the original Figure 7.

