# Author Correction: The influence of different sample preparation on mechanical properties of human iliotibial tract

**DOI:** 10.1038/s41598-021-89952-4

**Published:** 2021-05-11

**Authors:** Benjamin Fischer, Sascha Kurz, Andreas Höch, Stefan Schleifenbaum

**Affiliations:** 1 ZESBO - Center for Research On the Musculoskeletal System, Leipzig University, Semmelweisstraße 14, 04103 Leipzig, Germany; 2 Department of Orthopedic, Trauma and Plastic Surgery, Spine Center, Leipzig University, Leipzig, Germany; 3 Fraunhofer Institute for Machine Tools and Forming Technology, Chemnitz, Germany

Correction to: *Scientific Reports* https://doi.org/10.1038/s41598-020-71790-5, published online 09 September 2020

This Article contains errors. The determined values of the Young's moduli deviate from the literature by about two orders of magnitude. Due to the recalculation, the values of the determined Young’s moduli in Figure 7B are incorrect. The correct Figure 7B appears below as Figure 1.

**Figure 1:**
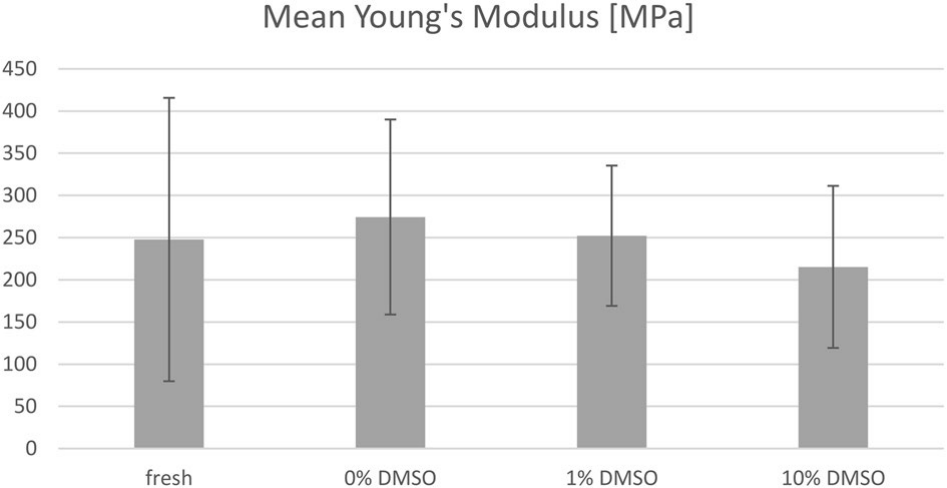
A correct version of the original Figure 7B.

In addition, a new table is included with each calculated Young’s modulus for better transparency. The calculated Young’s moduli are listed in Table 1 below.

**Table 1:** Determined Young’s moduli of the examined iliotibial tract specimens (5 donors, left and right side).

Sample	Young’s modulus (MPa)			
	Fresh	Fresh-frozen 0% DMSO	Fresh-frozen 1% DMSO	Fresh-frozen 10% DMSO
S1 Left	137.64	266.03	261.11	84.45
S1 Right	143.17	267.44	266.97	183.44
S2 Left	179.58	145.40	123.98	31.051
S2 Right	158.19	79.54	285.07	378.72
S3 Left	103.29	204.70	195.59	250.69
S3 Right	48.26	314.07	361.76	220.26
S4 Left	325.53	357.20	262.10	245.06
S4 Right	368.29	397.41	406.39	225.51
S5 Left	630.93	220.34	149.96	212.82
S5 Right	382.40	492.46	209.64	321.01
Mean	247.73	274.46	252.26	215.30
SD	167.91	115.60	83.16	96.04

As a result of this error, Table 2 is incorrect. The correct Table 2 appears below.

**Table 2:** A correct version of the original Table 2.

	F	FDF0	FDF1	FDF10
F_tu_ (MPa)	37.66 (SD 18.87)	34.64 (SD 12.08)	30.96 (SD 13.12)	22.00 (SD 10.91)
E (MPa)	247.73 (SD 167.91)	274.46 (SD 115.60)	252.26 (SD 83.16)	215.30 (SD 96.04)

In the Results section,

“A slight increase in the Young’s Modulus of FDF0 samples (0.91 (SD 0.29) MPa) and FDF1 samples (0.83 (SD 0.28) MPa), compared to F samples (0.73 (SD 0.44) MPa) has been observed (Fig. 7).”

should read:

“A slight increase in the Young’s Moduli of FDF0 samples (274.46 (SD 115.60) MPa) and FDF1 samples (252.26 (SD 83.16) MPa), compared to F samples (247.73 (SD 167.91) MPa) has been observed (Fig. 7).”

Finally, the Acknowledgements section is incomplete.

“We acknowledge support from the German Research Foundation (DFG) and Leipzig University within the program of Open Access Publishing.”

should read:

“We acknowledge support from the German Research Foundation (DFG) and Leipzig University within the program of Open Access Publishing. Additionally, the authors would like to thank Matthias Oehme and especially PD Dr. Hanno Steinke for their support in managing the cadaver acquisition.”

